# Enrichment of Druggable Conformations from Apo Protein Structures Using Cosolvent-Accelerated Molecular Dynamics

**DOI:** 10.3390/biology4020344

**Published:** 2015-04-21

**Authors:** Andrew Kalenkiewicz, Barry J. Grant, Chao-Yie Yang

**Affiliations:** 1 Department of Computational Medicine and Bioinformatics, University of Michigan, Ann Arbor, MI 48109, USA; E-Mail: ajkal@umich.edu; 2 Department of Internal Medicine, Hematology and Oncology Division, University of Michigan, Ann Arbor, MI 48109, USA

**Keywords:** Bcl-2 proteins, protein–protein interactions, principal component analysis, hierarchical clustering, binding site hotspot mapping, Sitemap, Bcl-xL inhibitors, computational docking

## Abstract

Here we describe the development of an improved workflow for utilizing experimental and simulated protein conformations in the structure-based design of inhibitors for anti-apoptotic Bcl-2 family proteins. Traditional structure-based approaches on similar targets are often constrained by the sparsity of available structures and difficulties in finding lead compounds that dock against flat, flexible protein-protein interaction surfaces. By employing computational docking of known small molecule inhibitors, we have demonstrated that structural ensembles derived from either accelerated MD (aMD) or MD in the presence of an organic cosolvent generally give better scores than those assessed from analogous conventional MD. Furthermore, conformations obtained from combined cosolvent aMD simulations started with the apo-Bcl-xL structure yielded better average and minimum docking scores for known binders than an ensemble of 72 experimental apo- and ligand-bound Bcl-xL structures. A detailed analysis of the simulated conformations indicates that the aMD effectively enhanced conformational sampling of the flexible helices flanking the main Bcl-xL binding groove, permitting the cosolvent acting as small ligands to penetrate more deeply into the binding pocket and shape ligand-bound conformations not evident in conventional simulations. We believe this approach could be useful for identifying inhibitors against other protein-protein interaction systems involving highly flexible binding sites, particularly for targets with less accumulated structural data.

## 1. Introduction

The Bcl-2 protein family constitutes a central class of regulators for the intrinsic apoptotic pathway [1]. The balance between pro- and anti-apoptotic members from this family is the major factor affecting mitochondrial outer membrane permeability, which induces cytochrome c release and leads to activation of downstream caspases [1]. Specific control of caspase activity in the cell by Bcl-2 family proteins is also required for differentiation and hematopoietic tissue homeostasis [2,3,4]. In many diseases, the apoptotic machinery in cells is dysfunctional and apoptosis becomes misregulated [5]. In cancer cells, overexpression of anti-apoptotic members can serve as a driving force for tumorigenesis or for acquisition of chemoresistance against drug treatments [6,7]. One widely pursued approach in apoptosis-based therapy entails developing chemical agents to inhibit the anti-apoptotic members of Bcl-2 proteins [5,8,9,10,11]. ABT-263 [12,13,14] and ABT-199 [15] are two potent inhibitors that have been successfully designed to target a subset of the anti-apoptotic Bcl-2 proteins, e.g. Bcl-xL, Bcl-2, and Bcl-w [16]. These two compounds are currently in late-stage clinical trials for treating myeloma, lymphoma, leukemia, and certain lung cancers [14,17].

Developing small molecules to inhibit the function of the anti-apoptotic members of the Bcl-2 proteins is challenging because it involves the inhibition of protein–protein interactions [18,19,20,21]. Anti-apoptotic Bcl-2 proteins have a long and flexible hydrophobic binding groove, which serves as a docking site for BH3 (Bcl-2 homology 3) domain-only proteins [22]. This protein–protein interaction prevents the latter from carrying out pro-apoptotic activities that would otherwise promote cell death [23]. Available crystal structures have revealed that the interaction between anti-apoptotic and pro-apoptotic members of the Bcl-2 proteins is mediated via four hydrophobic residues and one acidic residue of the pro-apoptotic members [24,25]. Using a “SAR-by-NMR” approach—followed by medicinal chemistry optimization targeting the anti-apoptotic Bcl-xL and Bcl-2 proteins—ABT-737 and ABT-199 were both developed from the same core scaffold [16]. Although many small molecule inhibitors have been reported to date [10,26,27], compounds with similar or higher potencies than ABT-737 and ABT-199 contain similar core structures and are all in early preclinical studies (e.g. TM-957 [28] and compounds based on quinazoline sulfonamides [29]). Furthermore, these compounds have very high binding affinities to Bcl-2, Bcl-xL, and Bcl-w, but not to other anti-apoptotic members such as Bcl2A1, Mcl-1, and Bcl-B. For example, ABT-737 binds to Bcl-2, Bcl-xL, and Bcl-w with a K_i_ of <1 nM, but has > 1000× weaker affinity to Mcl-1 and Bcl2A1 [16]. Because tumor cells can become resistant to ABT-737 or ABT-199 upon treatment by upregulating Mcl-1 and Bcl2A1 [30,31,32], next-generation small molecule inhibitors that target selectively Bcl2A1, Mcl-1 [33], and Bcl-B—or act as pan-Bcl-2 family inhibitors [34,35]—are highly desirable for future apoptosis-based cancer therapeutics [27].

Because the BH3-domain binding groove of anti-apoptotic Bcl-2 proteins is long and flexible, it can adopt multiple conformations and should in theory be able to bind ligands with substantially different physical topologies [22,36]. As mentioned before, however, there are currently only a handful of different scaffolds that have been developed into lead compounds with sufficient *in vivo* efficacy. This can be partly attributed to the limited degree of compound diversity in the small-molecule co-crystal structures that are available to use as the starting point for rational, structure-based drug design efforts. Additionally, no small-molecule co-crystal structures for Bcl2A1, Bcl-b, and Bcl-w have been reported to date. Despite their limitations, the co-crystal structures that are currently available can still be used as starting points for computational simulations that can potentially provide a much needed enrichment of conformations of the protein–protein interaction site.

Rational structure-based drug design efforts that aim to inhibit protein–protein interactions typically start with knowledge of a protein-protein or protein-peptide complex structure. The binding sites in these structures often conform to accommodate their relatively large binding partner. This results in non-optimal pocket conformations for small molecule binding, raising the question of whether these sites are druggable by small molecules. In such cases, the native ligand in the structure may be removed and molecular dynamics used to facilitate the sampling of conformations that are potentially more compatible to small molecule binding. However, this approach can limit the generation of larger exposed hydrophobic pockets due to unfavorable protein hydration. To assess druggability for PPI targets, a recent report proposed to carry out MD simulations with soluble organic cosolvent molecules [37]. In such simulations, the cosolvent molecules probe the interaction site and also help to reveal how the protein can be expected to respond when a generic small molecule ligand enters the binding site. Besides probing the binding site, the inclusion of cosolvent molecules in the system can also alter the population of protein conformations at equilibrium [38,39] and influence the dynamic transition rate of xylanase[40]. By employing these computational strategies, we have compared MD simulations starting from apo Bcl-xL in either a pure water or cosolvent environment and observed that the cosolvent simulations produced conformations with structural characteristics specific to known co-complex structures, while the pure water simulations did not [41]. One inherent challenge to our previous study is that the system may become trapped in energy minima, resulting in restricted conformational sampling across timescales common in conventional MD simulations. Accelerated molecular dynamics (aMD) offers a potential solution to this problem in that it utilizes a “boost potential” to essentially raise the energy wells and allow the system to overcome kinetic barriers more easily [42]. Compared to analogous conventional MD simulations, aMD has been shown to sample a larger range of protein conformational space, including an enhanced degree of sampling of small molecule binding hotspots [43].

In this work, we combined the aMD and cosolvent MD simulation methods to achieve efficient sampling from an apo-form protein in the presence of small cosolvent molecules acting as ligands. The anti-apoptotic Bcl-2 family member Bcl-xL was used as a test system because there is a relative abundance of small molecule co-complex structures available for Bcl-xL compared to other Bcl-2 family members. Conformations of one apo-form and one Bad BH3 peptide-bound Bcl-xL structure obtained from simulations using (a) pure water conventional MD, (b) cosolvent MD (with an isopropanol probe), (c) accelerated MD, (d) and cosolvent aMD were compared to the crystal structure conformations through principal component analysis (PCA). To assess the relative similarity between structures within a given simulation setting, we clustered the conformations from each trajectory in the subspace derived from the first and second principal components of the crystal structure PCA. Representative conformations were selected for a follow-up virtual screening evaluation—using 27 known small molecule inhibitors without reported co-crystal structures and 147 decoy compounds—to assess the small-molecule ligand binding capacity of our simulated conformations. Our results showed that the conformations of apo-form Bcl-xL in a cosolvent environment with accelerated MD yielded the greatest overall conformational variation in the experimental structure PC subspace. Structures obtained from this simulation setting also generally yielded more favorable docking scores for our entire set of small molecule compounds, suggesting that their associated binding site conformations are more adaptive to a wide range of small molecule ligands in virtual screening calculations. Taken together, the combination of aMD and cosolvent MD is an attractive approach for generating and enriching protein binding site conformations complementary to small molecule ligands, and can be useful for virtual screening studies aiming to identify novel ligands for subsequent biological evaluation. We believe this method should be particularly useful when only the apo-form crystal structure of a target protein is available, assuming the MD force field parameters can produce accurate conformational sampling. Our study suggests that the implementation of this approach would be helpful in identifying novel chemical leads to jump-start a small-molecule drug discovery program for other pro-apoptotic Bcl-2 family members such as Bcl2A1 or Bcl-w.

## 2. Experimental Section

All experimental and simulated structural data were analyzed with the Bio3D package [44]. The sequence of the crystallographic structure for human apo Bcl-xL (PDB code: 1MAZ) was used to BLAST [45] search the RCSB PDB for homologous structures. A total of 221 hits for the Bcl-2 family were reported, from which a sequence identity cutoff of 70% was used to isolate only Bcl-xL structures. This yielded a set of 56 unique PDB structures encompassing a total of 80 different chains. These structures include wild-type and mutant Bcl-xL from human, mouse, and rat. All are modified constructs with the membrane-binding C-terminal domain removed. Wild-type Bcl-xL protein contains a 60-residue loop between α1 and α2 that is absent in the majority of structures (a notable exception is PDB code: 1LXL, a solution NMR structure). It has been demonstrated in previous experiments that this loop is not required for anti-apoptotic activity [46].

The amino acid sequences of the 82 different chains were aligned using the MUSCLE algorithm [47]. Two of the chains (PDB code: 3IO8 chain A and 4HNJ chain B) were found to have significant gaps in key α3 residues, and hence these structures were omitted from subsequent analysis. All conformations were then structurally superimposed upon each other via least-squares-fitting of the Cartesian coordinates of equivalent C-α atoms from α2 and α5—as these two helices were found to be the most structurally invariant regions with respect to the BH3-domain binding groove. Three distinct folding patterns were identified in the overall set; 41 are globular monomers, 14 engage in α1 domain swapping, and two engage in α6/α8 domain swapping.

Principal component analysis (PCA) was used to assess inter-conformer relationships in the set of superimposed structures. This technique has previously been shown to be a valuable tool for assessing experimental structure distributions and comparing them to conformations obtained through MD simulations. In mathematical terms, PCA involves building and then diagonalizing a covariance matrix *C*, having elements *C_ij_*, from the Cartesian coordinates *r* of equivalent atoms from the superimposed experimental structures:

(1) $$C_{ij}=\langle \langle r_{i}-\langle r_{i}\rangle \rangle \text{∙}\langle r_{j}-\langle r_{j}\rangle \rangle \rangle$$

For N number of atoms, *i* and *j* are all the possible pairs of 3N Cartesian coordinates. The eigenvalues for the covariance matrix give the variance of the overall distribution along their respective eigenvectors, which themselves correspond to a linearized basis of the structural distribution and are also referred to as the “principal components” [48].

Both the experimental structures and MD trajectory frames were mapped into a subspace built from the dominant PCs to provide a lower dimensional representation of the original data. In this case, PCA was carried out on the Cartesian coordinates of C-α atoms from the region comprised of α2, α3, α4, α5, and all intervening loops. This roughly defines the region circumscribing the BH3-domain binding groove. It should be noted that α7 could also have been included here; however, the helix engages in domain-swapping in several structures from the experimental set. Since this study is primarily focused on the protein’s monomeric behavior, the helix was excluded from the PCA.

Regional flexibility in the set of experimental structures was compared to that seen in the MD trajectories by calculating residue-wise root-mean-squared fluctuations (RMSFs) for all C-α atoms. We also measured root-mean-squared deviation (RMSD) values for C-α atoms from individual helices in order to assess transitions between distinct structural states in the MD trajectories.

A series of conventional and accelerated molecular dynamics (MD) simulations were run using the Bad-bound (PDB code: 2BZW) and apo (PDB code: 1MAZ) crystal structures as starting conformations. The aMD method works to enhance conformational sampling by implementing a “boost” potential ΔV if the value of the potential energy is below a pre-specified threshold *E_b_*:

(2) $$V^{*}\left(\vec{r}\right)=V\left(\vec{r}\right),\;V\left(\vec{r}\right)\geq E_{b}$$

(3) $$V^{*}\left(\vec{r}\right)=V\left(\vec{r}\right)+\text{∆}V\left(\vec{r}\right),\;V\left(\vec{r}\right)<E_{b}$$

and

(4) $$\text{∆}V\left(\vec{r}\right)=\frac{{\left(E_{b}-V\left(\vec{r}\right)\right)}^{2}}{E_{b}-V\left(\vec{r}\right)+\alpha }$$

Here, the aMD threshold potential and acceleration parameter (α) were determined via the method used by Grant *et al.* [49].

All simulations were carried out using the CUDA implementation of the AMBER PMEMD method [50], with each individual simulation being run on a single NVIDIA GeForce GTX 680 GPU from the high-performance computing cluster at the University of Michigan Center for Computational Medicine & Bioinformatics. Simulations were run in either pure aqueous or 20% v/v isopropanol cosolvent solutions. Protonation states for ionizable groups were determined via the PROPKA method [51], and negative charges were neutralized using Na^+^ counter ions. For simulations carried out in pure water, the prepared protein structure was placed in a 13-Å octahedral water box using the TIP3P water model [52]. Cosolvent simulations entailed the use of a 13-Å octahedral cosolvent box of 20% v/v isopropanol in water, designed and supplied by Xavier Barril [37].

Minimization was carried out through 1000 steps of the conjugated gradient method followed by 2000 steps of steepest descent. The temperature of the system was raised from 0 to 298 K via 50 ps of simulation with constant volume/constant temperature in the canonical ensemble (NVT), with a 5 kcal/mol/Å^2^ force constraint on all backbone atoms. This was followed by 200 ps of simulation with constant pressure/constant temperature in the isothermal-isobaric ensemble (NPT), with a 2 kcal/mol/Å^2^ force constraint. Finally, 100 ns of production aMD were run under NPT conditions at 298K and 1 atm, with 2 fs time-steps. The SHAKE algorithm [53] was used to keep all hydrogen bonds fixed, and the cutoff for non-bonded interactions was set to 12 Å. Protocols for minimization and equilibration of the Bcl-xL cosolvent system were the same as those utilized by Yang and Wang [54].

We note that even with the relatively high van der Waal’s cutoff, as well as the additional calculations required for aMD, each 100 ns simulation of the 139 residue long Bcl-xL protein finished in approximately four days. This represents nearly a 19-fold increase in runtime efficiency over similar simulations run previously [41,54], which we attribute to using the CUDA implementation of PMEMD to carry out our simulations on NVIDIA GPUs.

Our grid-based hotspot mapping method was implemented as follows: the crystal structure of Bcl-xL/ABT-737 was aligned to the first conformation from the trajectories to extract ABT-737 as the reference ligand at the binding site. A 1-Å uniformly spaced grid was then set up with the origin at the center of mass of ABT-737. Grid points within 1.5 Å of the van der Waals’ radius of the protein atoms were removed. The remaining grid points within 3 Å of ABT-737 were considered to be within the binding site and saved for subsequent calculations. For the saved grid points, the “buriedness” of each grid point was calculated using a ray tracing method similar to Sitemap [55]. Specifically, 12 rays were emanated from each grid point, checking for any neighboring protein atoms in the binding site. If 10 of the 12 rays at a given grid point crossed any protein atom in the binding site, and the point was surrounded by more than three neighboring points with the same property, the grid point was considered “buried.” The latter of these two criteria allows for elimination of the boundary layer of grid points that are also highly exposed. The buried grid points were then saved, assigned the C.3 atom type, and evaluated for their interactions with the protein via M-Score [56]. After all the buried grid points were assigned scores, those grid points with scores better than one quarter of the lowest score were collected. If a grid point in this collection was also surrounded by more than four such grid points, it was designated as a “hotspot” grid point. When evaluating the hotspot grid points in an ensemble of conformations, all Bcl-xL conformations were aligned to the α5 helix of the apo-Bcl-xL crystal structure (PDB ID: 1MAZ), again using the center of mass of ABT-737 as the grid origin. By this setup, the same Cartesian coordinate system was used throughout the entire ensemble of Bcl-xL conformations in our evaluation.

## 3. Results and Discussion

### 3.1. Mapping of Bcl-xL Experimental Conformations Reveals Distinct Apo and a Diversity of Peptide and Inhibitor-Bound Forms

**Figure 1:**
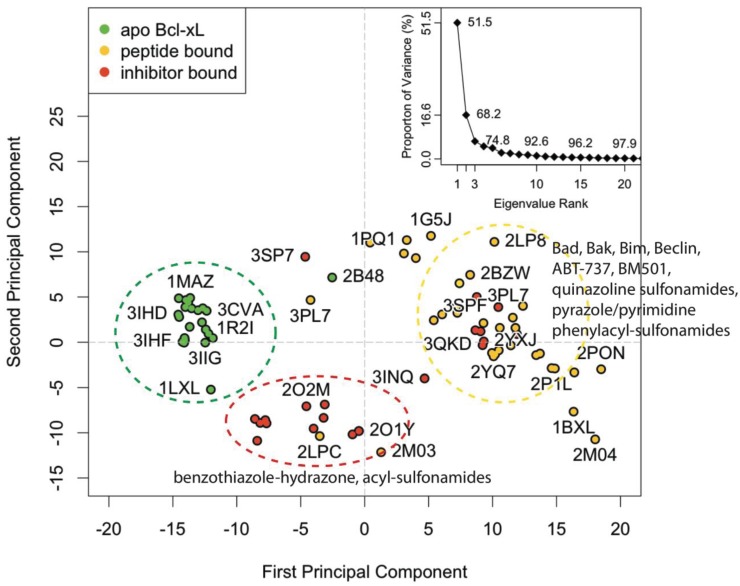
The PC subspace for the BH3-domain binding pocket of Bcl-xL experimental structures contains three general conformational groupings. The group furthest to the right (yellow circle) is dominated by peptide-bound conformations, in which α3 and α4 are shifted downward with respect to the apo structures (green circle). Meanwhile, the benzathiazole-hydrazone and acyl-sulfonamide inhibitor-bound structures (red circle) have an upward shifted α3 and α4 and a rightward shifted α3 with respect to the peptide-bound structures.

Principal component analysis was used to evaluate structural variation in the vicinity of the BH3 binding groove for all available Bcl-xL experimental structures (Figure 1; see Experimental Section for details). The first PC accounts for 51.5% of the total variance and corresponds to a coupled translational motion in the region comprised of α3, α4, and the α3–α4 loop. Our definition of alpha helix numbers follows previous reports [41]. In structures with more positive scores along PC1, the region is shifted upward, whereas in structures with more negative PC1 scores it is shifted downward. An additional 16.7% of the variance is captured by the second PC, for which positive and negative scores correspond to leftward and rightward longitudinal shifts in α3, respectively.

Nearly all of the known apo structures form a distinct structural subgroup that is clearly separated from the peptide-bound and inhibitor-bound structures (Figure 1, green points). A notable outlier is 2B48—an α6-α8 domain swapped dimer. Apart from this exception, the conformation of the BH3-domain binding groove region is nearly identical across the entire apo subgroup. Specifically, in contrast to other structures, α4 is bent upward, which facilitates a corresponding upward shift in α3 and allows the phenyl moiety from Phe105 to occupy the p2 pocket.

The holo (or non-apo) structures are distributed across a wider range of overlapping conformational space (Figure 1, yellow and red points). This reflects the significant diversity of peptides and ligands that are capable of docking in the BH3-domain binding groove. For the majority of these structures, α3 and α4 are shifted downward with respect to the apo structure, providing access to the hydrophobic pockets that mediate the interaction between protein and peptide or protein and ligand. PC1 and PC2 divide the holo set into two subgroups. The larger group contains most of the peptide-bound structures and a handful of ligand-bound co-complexes, including that for the well-known inhibitor ABT-737 (PDB code: 2YXJ). Structures from this group generally have more positive PC1 and PC2 values, representing conformations in which α3 and α4 are shifted downward and α3 is shifted leftward. The smaller group is comprised primarily of co-complexes associated with a special class of compounds containing a benzathiazole moiety, which binds in the p2 pocket of the BH3-domain binding groove. As a whole, the group is generally characterized by more negative values for both PCs, corresponding to conformations in which α3 and α4 are shifted upward while α3 is shifted longitudinally rightward.

Because the first two PCs account for a substantial majority of the conformational variation, we clustered the experimental structures in the PC1-PC2 subspace, allowing us to divide the set into 12 distinguishable subgroups. Roughly speaking, the benzathiazole-containing co-complexes comprise two of these clusters, and the rest of the ligand and peptide-bound structures are spread across another eight clusters. All but two of the apo structures fall into a single cluster. One of the outliers is the aforementioned domain-swapped dimer 2B48, while the other is the solution NMR structure 1LXL (which contains the full sequence of the Bcl-xL protein). The procedure for clustering the experimental structures was used to establish a similar protocol for clustering simulated conformations (see Experimental Section for details).

### 3.2. Conventional MD Captures Relaxation from Peptide-Bound to Apo-Like Conformations

**Figure 2:**
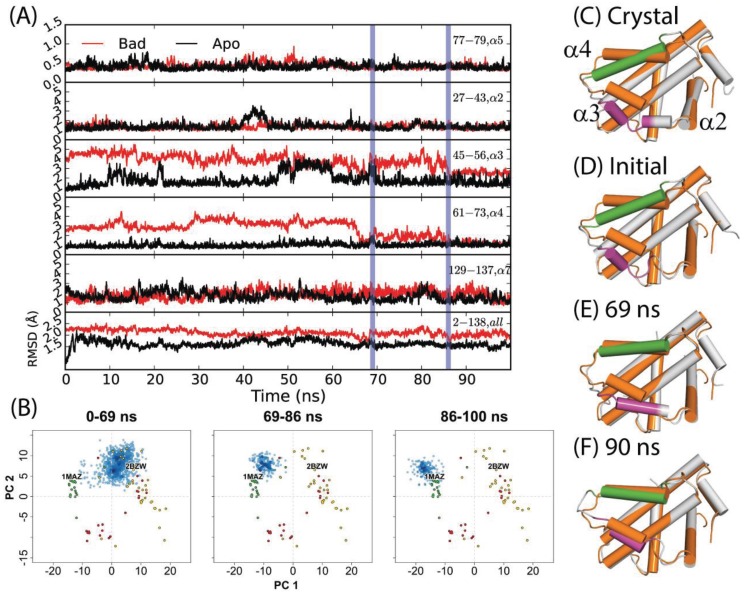
(**A**) RMSDs of α2, α3, α4, α5, α7, and the full-length protein for a 100 ns simulation starting from the Bad-bound structure (red) and the apo structure (black). The former saw a relaxation to an apo-like state after 86 ns, with key transition points indicated by vertical blue lines. (**B**) Specific portions of the trajectory corresponding to different time intervals were projected into the experimental structure PC subspace (blue points) for comparison to the RMSD analysis. (**C**) Crystal structures of apo- and Bad-bound Bcl-xL were aligned to depict the end states. Corresponding conformations at (**D**) 0 ns, (**E**) 69 ns, and (**F**) 90 ns are shown. For (**C**–**F**), the reference apo Bcl-xL structure is colored in orange. For the aligned conformations, the purple and green segments denote α3 and α4 helices along with other Bcl-xL segments colored grey.

Previously, Yang and Wang reported that a 50 ns simulation of holo-Bcl-xL/Bad (commencing from PDB code: 2BZW) in pure water did not sample the apo structure conformational space at any point [54]. For this study, we extended the simulation for another 50 ns in order to observe whether a longer timeframe would allow the protein to escape the holo-structure conformational space. Interestingly, the simulation yielded a relaxation to an apo-like state within the extended time period. An assessment of the time evolution of RMSD values to the canonical apo crystal structure (PDB code: 1MAZ) revealed that the transition occurred via three sequential steps (Figure 2D–F); first, α3 elongates, gaining one helical turn at its N-terminal end. This is reflected in a net RMSD decrease from approximately 5 Å to 4 Å over the first 30 ns. Next, α4 shifts sharply upward between 66 and 69 ns—yielding a decrease in RMSD from ~5 Å to 1 Å. The helix continues fluctuating upward and downward throughout the next 20 ns, with RMSD values varying from ~1–3 Å. Finally, α3 and α4 engage in a coupled upward shift at 86 ns, with the final α3 and α4 RMSDs dropping to ~2.7 Å and ~1.3 Å, respectively. The positions of α1, α2, and α5–α7 are relatively consistent between apo- and holo- Bcl-xL crystal structures, and the trajectory revealed only minor flexibility in these regions (Figure 2, Table S1). It should be noted that despite the dramatic decrease in α3 and α4 RMSD with respect to the 1MAZ PDB structure, the simulation did not fully reach the native apo conformation. In particular, the conformation of the α3–α4 loop in the final 14 ns of the trajectory differs substantially from the canonical apo structure, with an RMSD of over 6 Å.

The results of projecting the trajectory frames into the experimental structure PC1-PC2 subspace corroborated the conclusions drawn from the RMSD analysis (Figure 2B). As mentioned previously, PC1 represents an upward shift in α3 and α4. Because the initial refolding of α3 draws the overall α3 backbone slightly upward, most of the frames from the first 69 ns of the trajectory have slightly more negative PC1 scores than the starting structure. On a similar note, the sharp upward movement of α4 between 66 and 69 ns brings the trajectory frames even closer to the experimental apo structures along PC1. At 86 ns, the final coupled upward shift of α3 and α4 puts the trajectory frames just to the upper left of the apo set. Our observation that the trajectory did not fully reach the true native apo conformation is reflected here in the fact that the trajectory frames from the last 14 ns do not directly overlap with the apo crystal structures.

**Figure 3:**
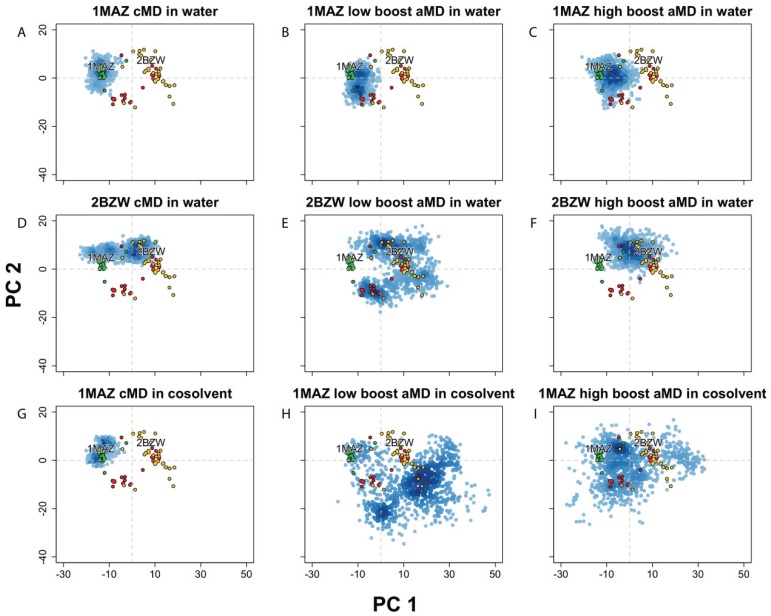
Projection of Bcl-xL conformations into the experimental structure PC1-PC2 subspace, from MD simulations using conventional MD (cMD) in water or a 20% v/v isopropanol/water cosolvent environment, along with accelerated MD (aMD) using low or high acceleration parameters. Simulations started with (**A**–**C**) apo-Bcl-xl (PDB code: 1MAZ) in water, with (**D**–**F**) Bad-bound Bcl-xL (PDB code: 2BZW) in water, and with (**G–I**) apo-Bcl-xl (PDB code: 1MAZ) in cosolvent environment. All simulations were run for 100 ns except for the 1MAZ cMD in cosolvent, which was run for 33 ns.

A 50 ns simulation of the Bcl-xL apo structure (PDB code: 1MAZ), also carried out by Yang and Wang [54], was reported to yield only minor deviations from the original crystal structure. As in the investigation of the holo-structure, we extended the apo simulation for another 50 nanoseconds. In this case, the extended portion resembled the initial 50 nanoseconds of the trajectory. Significant motions were restricted to α3 (Figure 2) and the α2–α3 loop (first half RMSD range: ~1–5 Å; second half RMSD range: ~1–4 Å). The simulation’s projection in the experimental structure PC subspace is consistent with these observations, with the trajectory frames being restricted to a small area centered on the apo crystal structures (Figure 3A).

### 3.3. Simulations with Enhanced Sampling Reveal Distinctive Conformational Landscapes

In order to further explore the dynamical characteristics of the Bcl-xL protein, we implemented several different techniques for enhancing conformational sampling on the apo- and holo-Bcl-xL crystal structures. First, we carried out two separate accelerated molecular dynamics (aMD) simulations on holo-Bcl-xL in a pure water environment, utilizing several different values for the aMD acceleration parameters. RMSD analysis and projection of the simulation snapshots into the crystal structure PC subspace revealed that both the “low boost” and “high boost” trajectories were able to generate extensive conformational sampling, although neither was able to sample the apo structure conformational space (Figure 3E–F). The high boost simulation exhibited the largest overall backbone motions (avg. RMSD: 4.71 ± 0.95), while the low boost simulation generated the largest fluctuations for α3 (avg. RMSD: 5.51 ± 2.44 Å). In the experimental structure PC subspace, the two trajectories had a similar degree of conformational sampling along PC1, whereas sampling along PC2 was greater for the low boost simulation (Figure 3E–F). Interestingly, in both trajectories, Phe105 becomes incorporated into α2 instead of into α3 as in the cMD simulation. Moreover, α2 and α3 end up combining into a single extended helix, though the effect is quite transient in the low boost simulation.

Simulations of apo-Bcl-xL in pure water using aMD also exhibited a fairly high degree of conformational variation, with the largest motions occurring in α3 for the low boost simulation (avg. RMSD: 3.89 ± 1.06 Å) and in α3 and α7 for the high boost simulation (avg. RMSDs: 3.13 ± 0.87 Å and 4.21 ± 1.44 Å). The trajectory projections cover a wider area in the experimental structure PC subspace than the apo cMD simulation, though the amount of overlap with the ligand and peptide-bound structures is limited (Figure 3B,C).

It has been noted previously that Bcl-xL simulations carried out in a cosolvent environment can enhance the protein’s conformational sampling. In particular, Yang and Wang [41] found that cosolvent simulations of apo- and holo-Bcl-xL were capable of generating conformations of the BH3-domain binding site that were not seen in analogous pure water simulations and exhibited features reminiscent of other cocrystal structures for Bcl-xL. However, the trajectory projection of the apo structure cosolvent simulation demonstrates that, like the cMD and aMD simulations in water, the extent of conformational sampling was limited to the area around the apo crystal structures (Figure 3G). We subsequently combined the aMD and cosolvent simulation methods in an attempt to facilitate wider conformational sampling of the apo structure. We found that the implementation of these enhanced sampling techniques in tandem can generate much greater motions than either one used separately, as the average backbone RMSD was far greater in the two cosolvent aMD simulations (5.61 ± 1.84 Å for low boost, 5.10 ± 1.86 Å for high boost) than any other simulation from the apo structure. Motions in α3 (5.61 ± 1.84 Å for low boost, 5.10 ± 1.86 Å for high boost) and α4 (5.61 ± 1.84 Å for low boost, 5.10 ± 1.86 Å for high boost) were particularly high, and the projection of the trajectories into the experimental structure PC subspace clearly demonstrates that the simulations were able to sample areas far beyond the apo crystal structures (Figure 3H–I).

In summary, the PCA results indicate that the simulations with enhanced sampling covered a large conformational space corresponding to a wide array of cocrystal forms. In the case of the apo structure, the combination of aMD and a cosolvent environment allowed the protein to achieve much wider sampling than using either method separately. This feature could be particularly helpful for systems where there may be a limited number of co-crystal structures available. We conclude that enhanced sampling MD simulations can be used to sample distinct structural landscapes that may not be readily accessible by traditional simulation methods, which may in turn facilitate the design of inhibitors for flexible protein targets with limited available experimental structure data.

### 3.4. Structural Ensemble Generation and Small Molecule Docking

Our next objective was to assess whether any of the conformations generated from the apo structure simulations were amenable to *in silico* docking against small molecule inhibitors. To achieve this, we compared docking scores for a set of known Bcl-xL inhibitors against five structural ensembles: one comprised of 72 experimentally determined Bcl-xL conformations, and the other four made up of trajectory conformations taken from each of the previously discussed apo-Bcl-xL simulations. To generate structural ensembles of a manageable size for small molecule docking, we first carried out hierarchical clustering on the full set of frames from each trajectory using the same protocols as for the crystal structure clustering. This yielded a total of nine clusters for the pure water cMD simulation, seven clusters for the cosolvent cMD simulation, 11 clusters for the pure water aMD simulation, and 64 clusters for the cosolvent aMD simulation. We then selected a single structural representative from each cluster by identifying their centroids, *i.e.* the conformations with the lowest RMSD to the average conformation of each respective cluster.

**Figure 4:**
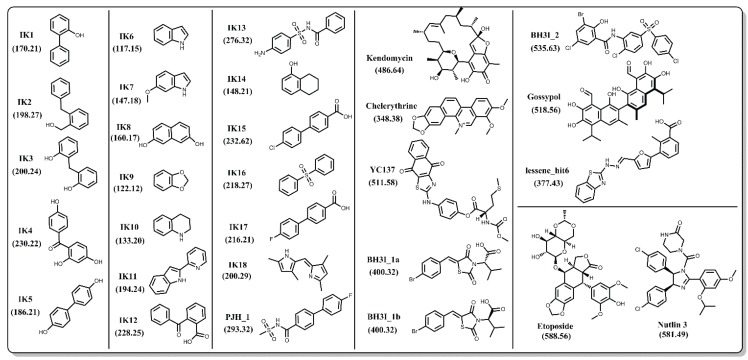
Chemical structures of 27 known inhibitors and two decoy compounds (Etoposide and Nutlin-3) selected for docking simulations. Numbers in parentheses are the molecular weights for each compound.

In order to achieve an unbiased comparison, we focused primarily on Bcl-xL inhibitors that do not have co-complex structures reported in the PDB. In the end, we selected a set of 27 natural and synthetic compounds of various sizes and chemical classifications (Figure 4), all of which have been reported to have inhibitory activity against Bcl-xL. Binding between Bcl-xL and the IK compound series was confirmed via 1D and 2D NMR spectroscopy [57,58,59]. PJH_1 is a fragment derived from ABT-737 and has a pK_D_ value of 3.5 [60]. Kendomycin [61], Chelerythrine [62], YC137 [63], BH3I_1a, BH3I_1b, BH3I_2 [64,65], and gossypol [66] were discovered by various research groups and their binding affinities to Bcl-xL have been confirmed by numerous reports including a recent study by Wan *et al.* [67]. Lessene_hit6 is a compound discovered by Lessene and coworkers [68] from a high-throughput screen and has a different scaffold from all other confirmed inhibitors. We note that several co-crystal structures with Bcl-xL have been determined for various derivatives of the original HTS hit (PDB codes: 3ZK6, 3ZLN, 3ZLO, 3ZLR). Repeated confirmed binding and structural data of these 27 compounds by different groups gives us confidence that their binding activity is not the result of assay artifacts.

**Figure 5:**
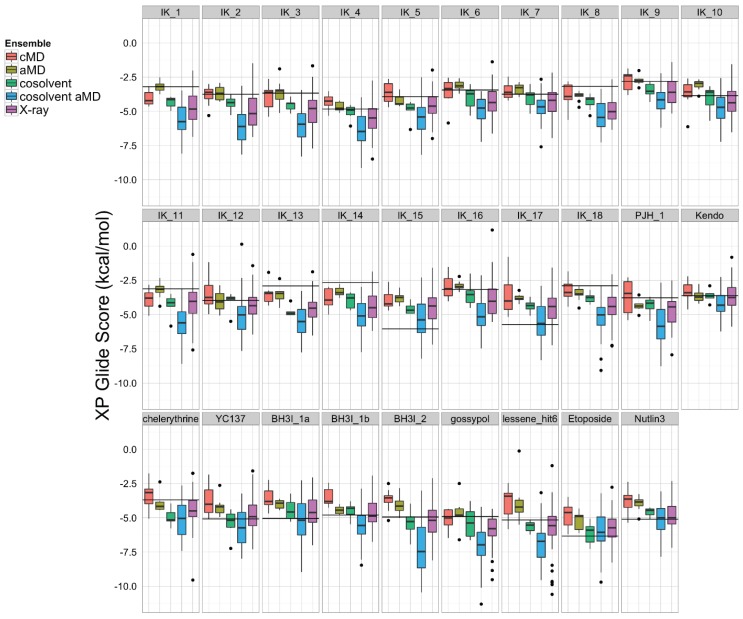
A set of 27 known Bcl-xL inhibitors and two decoy compounds were docked against the simulated and experimental structure ensembles. The distributions of docking scores for each ligand (individual panels) from each ensemble are shown as box-and-whisker plots with outliers as black dots. Docking with the cosolvent aMD (blue)-derived conformations yielded the best overall scores. The horizontal black line in each panel denotes the score achieved by docking against the single X-ray apo structure from which simulations were initiated (see Experimental Section for details).

To assess which structural ensemble had the best overall conformations for *in silico* docking, we used the Schrödinger Glide [69] utility to dock the 27 ligands from the aforementioned small molecule set against our five structural ensembles, and then performed a comparative analysis of the resulting docking score distributions (Figure 5). In general, simulations with a greater degree of enhanced sampling tended towards better docking scores. Interestingly, the structural ensemble taken from the apo-Bcl-xL cosolvent aMD simulation repeatedly achieved better docking scores than any of the other ensembles—including the experimental structures—for virtually the entire ligand set. In fact, the median Glide docking score for the cosolvent aMD ensemble was lower than the median docking score for the crystal structure ensemble for all 27 members of our small molecule set, and the minimum score from the cosolvent aMD set was better than the minimum score for the crystal structures for 25 out of the 27 ligands. Of the two exceptions, one of these is the small molecule inhibitor identified by Lessene *et al.* [68]. Not surprisingly, we found that the members of the experimental structure ensemble that scored better than the cosolvent aMD were the structures from the Lessene hit co-complex itself (PDB codes: 3ZK6, 3ZLN, 3ZLO, 3ZLR). The other exception was the natural compound chelerythrine, which docked best against the crystal structure for PUMA-bound Bcl-xL (PDB code: 2M04).

We found, furthermore, that the average interquartile range of docking scores for the cosolvent aMD cluster representatives (1.74 kcal/mol) was larger than that for the experimental structure set (1.45 kcal/mol), whereas the average interquartile range of docking scores for the smaller ensembles was significantly reduced (pure water cMD: 0.52 kcal/mol; pure water aMD: 1.10 kcal/mol; cosolvent cMD: 0.68 kcal/mol). This observation, along with our analysis of the ligand-specific median and minimum ensemble docking scores, provides evidence that cosolvent aMD simulations can be used to generate a structural ensemble with characteristics similar to a large set of experimentally determined structures, as far as docking-related properties are concerned.

The encouraging results of our docking simulations using the set of 27 known inhibitors motivated us to investigate the extent of general promiscuity of the binding site by screening against additional decoy compounds. We specifically included two non-binding compounds, etoposide and nutlin-3, used by Wan *et al.* [67], in addition to 145 fragment molecules provided by Dr. Isabelle Krimm—none of which were reported to bind Bcl-xL based on NMR fragment screening experiments [70]. Docking scores of these decoy molecules to our five structural ensembles exhibited similar trends to those of known inhibitors, *i.e.*, the cosolvent aMD simulations gave better or comparable scores than those from the experimental Bcl-xL structures (Figure 5 and Figure S1). Many of the decoy compounds also gave a higher score value (weaker affinities) than known inhibitors of similar molecular weights (etoposide and nutlin-3 *vs.* gossypol and BH3I_2 in Figure 5). This reaffirmed the structural adaptability of the cosolvent aMD conformations in binding to small molecule ligands. Although we expect there would be hit enrichment using the cosolvent aMD Bcl-xL conformations in a larger scale *in silico* virtual screen, follow-up docking studies would have to be used to assess the overall suitability of these conformations for identifying useful hits. Future work will entail a detailed investigation of the effects of docking engine and scoring function choice on enrichment for known inhibitors.

### 3.5. Comparison of the Experimental and MD Generated Binding Site Conformations Using Grid-Based Hotspot Mapping and Sitemap Analysis

**Figure 6:**
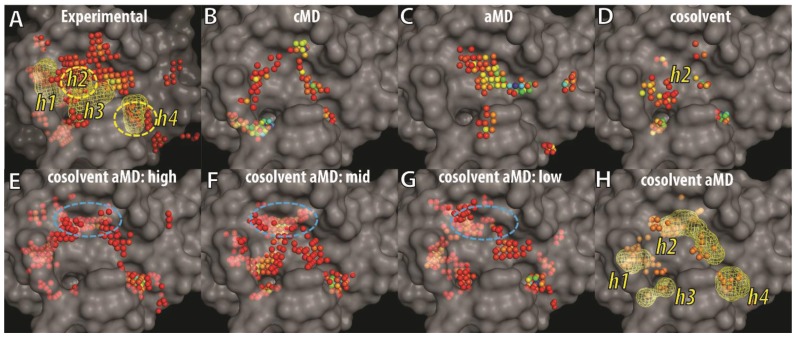
Grid-based hotspot mapping of (**A**) 72 experimental Bcl-xL structures and representative conformations selected by hierarchical clustering using the trajectories of (**B**) cMD; (**C**) aMD; (**D**) cosolvent MD; or (**E**–**H**) cosolvent aMD simulations, starting from the apo-Bcl-xL structure (PDB ID: 1MAZ). The representative conformations selected in (**E**–**G**) are the cluster groups with the most (**E**) to the least (**G**) number of members. Grid points showing up more than twice in (**E**–**G**) are shown as the mesh shape and orange points in (**H**). Pockets that interact with four key hydrophobic residues from the BH3 peptide—such as that in the Bad protein—were shown in mesh envelopes and labeled as h1–h4 in (**A**). The color of the hotspot grid points in (**A**–**G**) from blue to red correspond to high or low score values as assessed by the M-Score function. The transparent surface of the apo-Bcl-xL structure was used as a reference to illustrate buried pockets identified in other Bcl-xL conformations.

To visualize and differentiate between the binding site conformations of different ensembles of Bcl-xL conformations in our docking evaluation, we implemented a grid-based hotspot mapping analysis. This method assesses the preferences of probe atoms positioned at uniformly-spaced grid points in the binding site of a protein conformation, based on scores calculated from the knowledge-based scoring function M-Score [56]. The higher the scores, the more favorable the interaction is between the protein and the probe at the specified position. Probe atoms at grid points with scores satisfying pre-defined criteria were classified as hotspot grid points (see Experimental Section). Because the BH3 peptide binds to the BH3-domain binding-site of Bcl-xL primarily via hydrophobic interactions, an atom with the C.3 (saturated carbon) type was used as the probe atom. To focus on the most important regions of the binding site, we selected hotspot grid points with scores lower than the fourth quantile of all hotspot scores in each ensemble of Bcl-xL conformations. The four consensus sites (h1–h4)—derived from the interacting hydrophobic residues of the BH3 peptide—were used as location references in the binding site (Figure 6A). Mapping of hotspot grid points obtained from all experimental Bcl-xL structures showed that in the apo state, the h3 and h4 sites are exposed and preformed, whereas the h2 and h1 sites are semi-exposed or completely buried. Conformational flexibility of the α3 and α4 helices facilitates binding site remodeling at the h1 and h2 sites for Bcl-xL, allowing it to bind to different ligands. The analysis also mapped a large cluster of hotspot grid points at a site below h3 that was recently shown to interact with the p53 DNA-binding domain in a Bcl-xL and p53 crystal structure complex [71]. In the ensembles of cMD and aMD conformations, the h4 site was more likely to be characterized as a hotspot while the pockets at the h1 and h2 sites were not well formed or buried. In contrast, hotspot maps for the ensemble of cosolvent MD generated conformations showed that a deeper pocket at the h2 site was induced. In summary, the analysis indicated that ligand-induced binding pockets at the h1 and h2 sites were not well represented by either cMD or aMD sampling in aqueous conditions, but were partly captured in the cosolvent MD simulation.

Next, we analyzed the binding site conformations from the ensemble of cosolvent aMD structures, further dividing the set into three subgroups because of their broader conformational diversity (Figure 3). Based on the number of trajectory frames from each designated cluster (which ranged from 49 to 1), the subgroups were denoted as high-, middle-, or low-population clusters—resulting in three groups containing 22, 20, and 22 conformations, respectively. In addition to the pockets discussed previously (Figure 6B–D), several other hydrophobic binding pockets were identified. They include a location buried in apo-Bcl-xL at the h1 site and another more deeply penetrating location close to the h2 site (blue circle in Figure 6E–G). The h4 site was clearly a well-defined pocket in all 64 conformations shown in Figure 6E–G. In Figure 6H, we further selected the hotspot grid points appearing either two or three times in Figure 6E–G to highlight the recurrent hotspot grid points in the 64 conformations. Most of them fell into the mesh shapes representing hydrophobic residues of the BH3 peptide (see Figure 6A). These results suggest that the ligand-induced binding pockets at the h1 and h2 sites can be easily identified from the 64 conformations obtained from the cosolvent aMD simulation.

**Figure 7:**
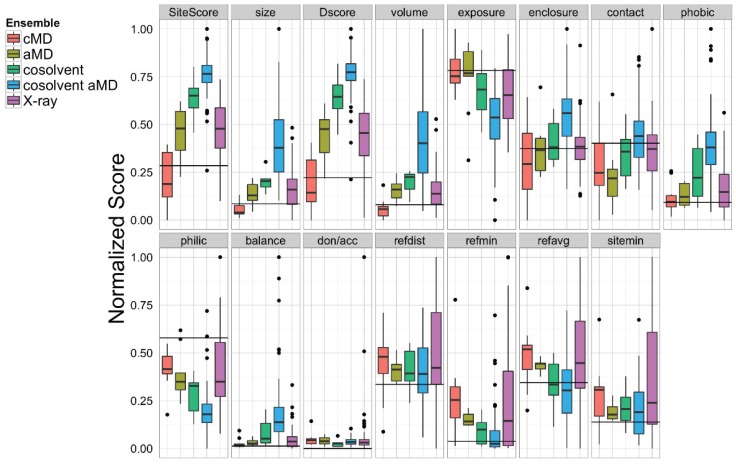
Sitemap analysis of the BH3-domain binding site in the ensemble of Bcl-xL conformations obtained from cMD, aMD low boost (aMD), cosolvent, cosolvent low boost aMD, and the experimental structures. The horizontal black line in each panel denotes the values calculated from the single X-ray apo Bcl-xL structure.

Apart from our novel grid-based hotspot mapping analysis, we also used the Schrödinger Sitemap [55] utility to further analyze the Bcl-xL conformations. Sitemap has previously been applied to analyze protein-ligand co-crystal structures with known ligand binding affinities to provide a metric for assessing binding site druggability [55]. Based on previous studies, binding sites with Dscore values < 0.83 were considered undruggable or very challenging. Our Sitemap analysis on the BH3-domain binding sites in 72 Bcl-xL crystal structures gave an average Dscore value of 0.91, suggesting that it is a moderately challenging binding site (Figure 7). Of all five ensembles, the cosolvent aMD simulation gave the highest maximum Dscore value (1.43, compared to 1.06 for pure water cMD, 0.87 for pure water aMD, 1.26 for cosolvent cMD, and 1.18 for the experimental structures). A similar trend was found for the Sitescore values, which are known to be more useful for identifying binding sites in an overall protein structure. The sizes of the binding sites from the cosolvent aMD-generated conformations were also larger but more enclosed than those of the crystal structures. In addition, the exposure and enclosure of the binding sites in the cosolvent aMD conformations are closer to the values of the submicromolar sites (average exposure/enclosure = 0.52/0.76) analyzed previously by Halgren [55]. We conclude that the quantitative analysis from Sitemap is consistent with the results of our novel grid-based hotspot mapping analysis (Figure 6E–H).

## 4. Conclusions

Proteins involved in protein–protein interactions have traditionally been considered difficult targets for small molecule inhibitor development. A recent review by Arkin *et al.* summarized progress in the field, including several inhibitors that are in clinical trials—providing strong evidence that this difficult class of protein targets is still tractable for therapeutic development [20]. The anti-apoptotic members of the Bcl-2 family, such as Bcl-xL, are one relatively successful example. The challenge in developing inhibitors targeting Bcl-xL has been attributed to the long hydrophobic BH3 domain binding groove, flanked by two inherently flexible helices forming the binding pocket—as described in this study.

In this work, we have combined the recently developed accelerated MD method (which enhances sampling of binding site conformations) with the cosolvent MD simulation method, which allows small organic cosolvent molecules to probe and reshape the Bcl-xL binding site. Principal component analysis confirmed that the approach samples a wide range of the crystal structure conformational space, and that our simulation of the Bad-bound Bcl-xL conformation in water induced relaxation to the apo state. The diverse Bcl-xL conformations obtained from the cosolvent aMD simulation were further analyzed via hierarchical clustering to select representative conformations. Using 27 known inhibitors ranging from fragment molecules to a large macrocyclic compound (Kendomycin)—with molecular weights from 117 to 535—in our *in silico* screening evaluation, we have shown that the ensemble of Bcl-xL conformations obtained from the cosolvent aMD simulation gave better median scores than ensembles of Bcl-xL conformations obtained using different simulation protocols or from experimental structures. These more favorable docking scores would suggest a better complementary fit between the small molecule inhibitors and the Bcl-xL conformations.

Analyses of the Bcl-xL conformations using our grid-based mapping method and the Sitemap program revealed that buried binding pockets at two locations (*i.e.*, h1 and h2 sites) can be well-characterized in the ensemble of Bcl-xL conformations from the cosolvent aMD simulations, but not for the conformations obtained from cMD and aMD simulations in aqueous conditions. These two buried pockets can be readily identified in several ligand-bound Bcl-xL experimental structures. An important point regarding our ensemble of Bcl-xL conformations obtained from the cosolvent aMD simulations is that we used the apo form of Bcl-xL as the starting conformation. In relation to our previous report that cosolvent molecules can promote and stabilize Bcl-xL conformations resembling ligand-bound structures, we found that the cosolvent aMD simulation also allowed greater conformational sampling of these types of conformations than a conventional cosolvent MD simulation. Thus, the cosolvent aMD method is an attractive sampling approach for enrichment of binding site conformations that are suitable for an *in silico* screening campaign using small molecules. We plan to carry out a detailed assessment of the Bcl-xL conformations obtained from our cosolvent aMD simulations, with the ultimate goal of achieving hit enrichment in a larger *in silico* screening campaign. This analysis will also include a comparison of different docking engines, the inclusion of a larger decoy compound dataset [72], and the exploration of experimentally validated inhibitors of Bcl-xL.

In summary, we conclude that this approach could be useful for investigating other targets with large protein–protein binding interfaces, particularly in cases where only an apo form crystal structure is available. Although our study was aided by the relative abundance of high-resolution Bcl-xL crystal structures, it stands as proof-of-concept for the overall approach, and certainly the most promising application of cosolvent aMD simulations would be for targets with less accumulated structural data. Nevertheless, a recent study addressing the validity of using long-timescale molecular dynamics simulations to refine protein homology models suggested there might be substantial limitations in the use of current force field parameters for characterizing conformations substantially away from native states in other protein systems [73]. We are currently investigating the applicability of our method to other protein targets outside the Bcl-2 family. These studies will provide more insight on the ways in which our approach can be generalized to help identify inhibitors for other important but challenging protein-protein interaction targets.

## Supplementary Files

Supplementary File 1
