# Supplementary material for: Enrichment of Druggable Conformations from Apo Protein Structures Using Cosolvent-Accelerated Molecular Dynamics
Source: Biology (Basel). 2015 Apr 21;4(2):344–66. doi: 10.3390/biology4020344 (PMC4498304; doi:10.3390/biology4020344)
Supplement: Supplementary File 1 [file biology-04-00344-s001.pdf]

Supplemental Materials

Table S1. RMSDs of the backbone atoms in different segments of Bcl-xL in the simulations using different parameters with reference to the apo-Bcl-xL crystal structure (PDBID: 1MAZ).

**Simulation System**	**α1 (4–18)**	**α2 (86–98)**	**α3 (104–111)**	**α4 (119–131)**
1MAZ cMD	1.65 ±0.38	1.24 ±0.21	2.23 ±0.86	1.84 ±0.48
1MAZ low boost	1.94 ±0.81	1.28 ±0.25	3.89 ±1.06	1.95 ±0.65
1MAZ high boost	2.10 ±0.37	1.45 ±0.25	3.13 ±0.87	2.72 ±0.72
2BZW cMD	1.75 ±0.34	0.82 ±0.21	2.83 ±1.00	2.39 ±1.44
2BZW low boost	2.77 ±1.19	1.43 ±0.46	5.51 ±2.44	2.57 ±0.90
2BZW high boost	5.29 ±2.44	1.56 ±0.34	4.24 ±0.95	3.62 ±1.01
1MAZ cosolvent cMD	1.82 ±0.44	1.40 ±0.18	2.06 ±0.67	1.73 ±0.55
1MAZ cosolvent low boost	2.73 ±0.87	0.95 ±0.25	7.86 ±2.13	8.61 ±3.58
1MAZ cosolvent high boost	2.76 ±1.42	1.83 ±1.39	6.57 ±1.88	4.96 ±2.18
2BZW cosolvent cMD	1.08 ±0.29	0.67 ±0.14	3.60 ±1.40	1.75 ±0.67
2BZW cosolvent low boost	3.68 ±1.75	1.98 ±0.83	7.73 ±1.77	5.39 ±2.91
2BZW cosolvent high boost	2.46 ±0.78	1.14 ±0.40	8.38 ±3.67	7.25 ±3.43
Simulation System	α5 (137–156)	α6 (162–176)	α7 (188–192)	all (1–196)
1MAZ cMD	0.78 ±0.11	1.64 ±0.40	2.23 ±0.54	1.99 ±0.27
1MAZ low boost	0.78 ±0.17	1.30 ±0.44	1.84 ±0.60	2.23 ±0.30
1MAZ high boost	1.07 ±0.19	1.85 ±0.63	4.21 ±1.44	3.33 ±0.51
2BZW cMD	0.68 ±0.12	1.10 ±0.34	1.14 ±0.58	1.97 ±0.56
2BZW low boost	0.92 ±0.19	1.34 ±0.57	1.69 ±0.92	2.68 ±0.65
2BZW high boost	1.85 ±0.57	3.73 ±1.74	4.69 ±1.59	4.71 ±1.00
1MAZ cosolvent cMD	0.70 ±0.16	1.79 ±0.44	1.60 ±0.56	2.05 ±0.31
1MAZ cosolvent low boost	1.44 ±0.28	3.87 ±3.08	3.33 ±1.69	5.61 ±1.84
1MAZ cosolvent high boost	1.96 ±1.06	3.42 ±2.08	5.70 ±3.66	5.10 ±1.86
2BZW cosolvent cMD	0.68 ±0.10	1.15 ±0.39	1.61 ±0.50	1.95 ±0.46
2BZW cosolvent low boost	1.92 ±0.85	3.78 ±1.85	8.97 ±5.09	5.64 ±1.94
2BZW cosolvent high boost	1.26 ±0.38	2.85 ±1.04	5.17 ±3.08	5.51 ±1.73

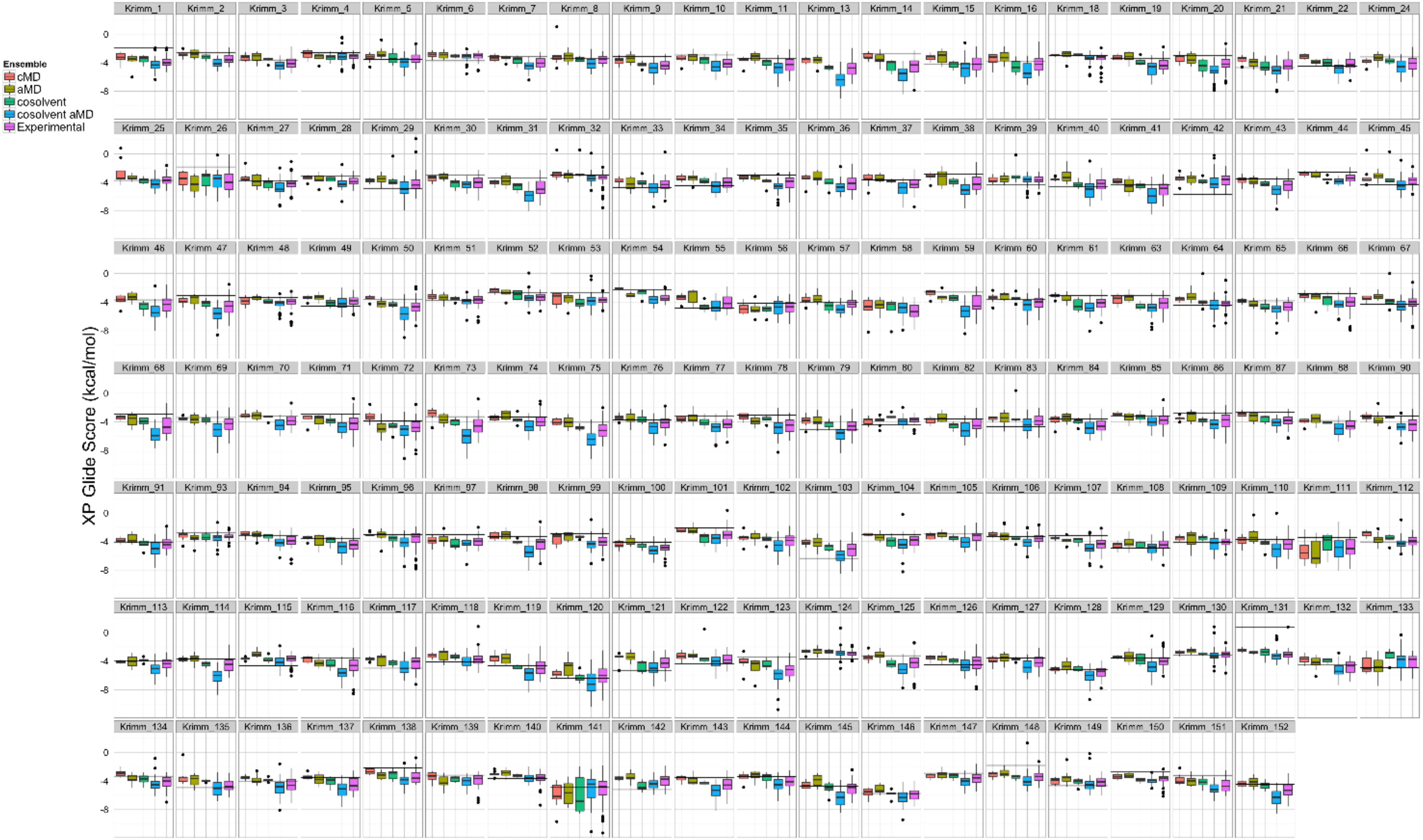

Figure S1. 145 decoys docked against the simulated and experimental structure ensembles.
